# A New Pooling Approach Based on Zeckendorf’s Theorem for Texture Transfer Information

**DOI:** 10.3390/e23030279

**Published:** 2021-02-25

**Authors:** Vincent Vigneron, Hichem Maaref, Tahir Q. Syed

**Affiliations:** 1 Computer Science Department, Univ Evry, Université Paris-Saclay, 91190 Saint-Aubin, France; hichem.maaref@univ-evry.fr; 2 School of Applied Sciences (FCA/UNICAMP), Limeira, Sao Paolo 13484-350, Brazil; 3 Computer Sciences Department, Institute of Business Administration, Karachi, Sindh 75270, Pakistan; tqsyed@iba.edu.pk

**Keywords:** deep learning, pooling function, Zeckendorf theorem, Fibonacci, LBP, image representation, segmentation, glioblastoma

## Abstract

The pooling layer is at the heart of every convolutional neural network (CNN) contributing to the invariance of data variation. This paper proposes a pooling method based on Zeckendorf’s number series. The maximum pooling layers are replaced with Z pooling layer, which capture texels from input images, convolution layers, etc. It is shown that Z pooling properties are better adapted to segmentation tasks than other pooling functions. The method was evaluated on a traditional image segmentation task and on a dense labeling task carried out with a series of deep learning architectures in which the usual maximum pooling layers were altered to use the proposed pooling mechanism. Not only does it arbitrarily increase the receptive field in a parameterless fashion but it can better tolerate rotations since the pooling layers are independent of the geometric arrangement or sizes of the image regions. Different combinations of pooling operations produce images capable of emphasizing low/high frequencies, extract ultrametric contours, etc.

## 1. Introduction

Deep neural networks (DNN) have revolutionized orthodox tasks of image analysis in which they have accomplished outstanding results and continually do so [1,2,3]. By employing modifications to the architectures and introducing various techniques (often greedy), considerable improvements have been achieved.

Convolutional Neural Networks (CNNs) architectures are increased through multiresolution (pyramidal) structures, which come from an idea that the network needs to see different levels of detail to produce good results. A CNN stacks four different processing layers: convolution, pooling, and fully connected (dense) layers [4].

The pooling layer receives multiple feature maps from convolutional layers and applies the pooling function to each of them. The pooling layer (a) reduces the number of parameters in the model (subsampling) and calculations in the network while preserving their important characteristics, (b) improves the efficiency of the network and prevents overlearning [4]. To do this, the maximum pooling function downsamples the input representation by reducing its dimensionality: the image is split into regular cells without overlapping, then the maximum value is kept within each cell. Thus, the pooling layer makes the network less sensitive to the position of features: the fact that a feature value is a little higher or lower, or even that it has a slightly different orientation *should not* lead to a drastic change in the image classification.

The weaknesses of pooling functions are well identified [5]: (a) they do not preserve all the spatial information well by reducing the spatial resolution, (b) the discrete maximum chosen by the maximum pooling in the pixel grid may be not the true maximum, (c) average pooling assumes a single mode with a single centroid. Hence, the question is how to take into account in an optimal way the characteristics of the input image being grouped in the pooling operation [6]? Part of the response sets in Lazebnik’s work, which demonstrated the importance of the spatial structure of pooling neighborhoods [7]. These local spatial variations of image pixel intensities (named textures in popular image processing) characterize an “organized area phenomenon” [8], which cannot be captured in pooling layers.

This paper proposes a new pooling operation, independent of the geometric arrangement or sizes of image regions, which can therefore better tolerate rotations. The operation is based on the Zeckendorf theorem for the decomposition of integers, which is also simple to implement. Zeckendorf theorem is mainly used in cryptography [9], e.g. to design small microcontrollers that can resist certain Fault Attack.

The rest of article is organized as follows: Section 2 presents the related works on pooling strategies. Zeckendorf additive partition is exposed in Section 3 and its implementation is explained in Section 4. Numerical experimentations and results are presented in Section 5. Finally, experimental works are discussed and future works are mentioned in Section 6.

## 2. Related Works

Throughout this paper small Latin letters a,b,⋯ represent integers. small bold letters a,b are put for vectors and capital letters A,B for matrices or tensor depending of the context. ‘{⋯}’ brackets indicate set of values. |·| is put for the cardinal operator.

### 2.1. Pooling Strategies in Image Processing

Convolutions in CNNs are discrete convolutions of an image *V* with a kernel *K*. Without loss of generality an input image *V* in a high dimensional space can be reduced into a vector v. Let’s define N(i) as the set of all indices of elements in v which are neighbors of $v_{i}$ in the neighborhood defined by the convolution kernel *K*

(1) $$N\left(i\right)=\left\{j\in N|v_{j}\in \;\mathrm{neighborhood}\;\mathrm{of}\;v_{i}\;\mathrm{given}\;\mathrm{by}\;K\right\}$$

As the structure of the neighborhood is fixed, we assume that N(i,j)∈{1,2,⋯,|N(i)|}, which is the index of *j* in N(i,j). The discrete convolution can then be defined as

(2) $$c{\left(k,v\right)}_{i}=\sum_{j\in N(i)}k_{N(i,j)}v_{j}.$$

where k are the the weights of the convolution kernel *K*.

The exponential growth of the number of parameters makes convolutions with large kernel sizes computationally expensive. Therefore, most CNN architectures keep the kernel size at 3 × 3 or 5 × 5. However, how does one do a sensitive prediction for an entire image, if a single convolution “sees” only a 3 × 3 neighborhood? The solution is the stacking of convolutional layers. With two layers following each other, the last one can “see” a 4 × 4 neighborhood. This means a lot of convolutions must be stacked to have a receptive field as large as a reasonable input image. The increase in receptive field by convolution can be considerably higher when the image is downsampled to a lower resolution between two convolution operations. Various methods exist for resampling a given feature layer at multiple rates prior to convolution such as dilated convolution that “inflate” the kernel by inserting holes between the kernel elements [10] or astrous convolution [11].

Maximum pooling is a popular choice for this downsampling operation. The pooling operations have been little revised beyond the main current maximum, average, and stochastic pooling options despite indications that choosing multiple pooling functions can improve performance [12].

Sharma et al. analyzed and discussed qualitatively the performances of pooling strategies on different datasets [13]. Lee et al. [6] experimentally demonstrate that their pooling operations combining maximum and average pooling provide an increase in invariance properties over the conventional pooling. Lee et al. proposed to combine pooling filters that are themselves learned. In [14], Gulcehre et al. investigate a novel nonlinear unit, called $L_{p}$ unit that generalizes a number of conventional pooling operators such as mean, root mean square, and maximum pooling.

Agostinelli learned activation functions to improve DNN in [15]. Boureau et al. analyze theoretically why max pooling works well in a wide variety of contexts, even if similar or different factors come into play in each case [16].

Many researchers are working on the development of advanced pooling mechanisms to effectively use these essential features of pooling [13], in particular on how to bring learning to the characteristics of the region being pooled into the pooling operation [6]?

### 2.2. Pooling and Statistics

In Statistics, “pooling” describes the practice of bringing together small datasets that are assumed to have the same value of a characteristic, e.g., a mean, and using the larger combined set (the “pool”) to get a more precise estimate of this feature. *Poolability* can be formulated on the basis of the concept of statistical equivalence. Sheskin compiled in [17] a bibliography dealing with pooling procedures, for example to combine several independent tests of the same hypothesis.

The goal of pooling is to transform the convolutional characteristics into a new representation that preserves important information while ignoring irrelevant details. For instance, if a *t*-test between the two within-group slopes is not “passed”, these characteristics cannot be grouped [18].

In some way, many other ensemble techniques, where a set of weak learners are combined to create a stronger learner, are very near to this notion of pooling [19].

So, should we pool or not? Or, putting it a little differently, when should we pool and when should we not? The answer depends on the training context. Moorthy et al. in [20] proposed to weight the image quality measures by visual importance to improve the correlations with subjective judgment. Achieving invariance to changes in position or lighting conditions, robustness to size, and compactness of representation are all common goals of pooling. We demonstrate experimentally here that these properties are achieved successively with the Z pooling operator, based on Zeckendorf number theorem.

Experimental validation is continued in Section 5 on predefined architectures and obtained by replacing the standard pooling operations with Z pooling.

### 2.3. Texture Coding

**Figure 1:**
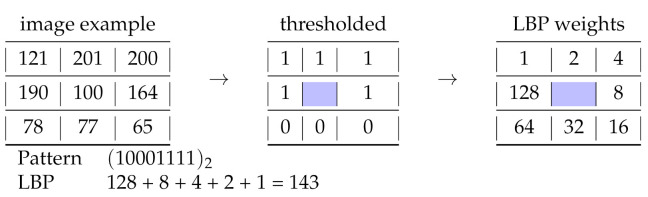
Example of 3 × 3 image neighborhood (P=8 and R=1).

Most of image descriptors that encode local structures e.g., local binary patterns (LBP) (and its variants) [21,22] depend on (a) the size of the neighborhood, (b) the reading order of the neighbors, (c) the mathematical function that is used to compute the feature distance between neighboring pixels. The new pixel value $L_{R}\left(P\right)$ in the image is an integer in the range of 0 to 255 (for a 8-bit encoding) given by:

(3) $$L_{R}\left(P\right)=\sum_{p=0}^{P-1}2^{p}\;\cdot \;t\left(g_{p}-g_{c}\right),\;\mathrm{with}\;t\left(x\right)=\left\{\begin{array}{cc} 1 & \mathrm{if}\;x\geq 0\\ 0 & \mathrm{otherwise} \end{array}\right.,$$

where *P* is the number of pixels in the neighborhood considering the distance *R* between central pixel $g_{c}$ and the neighboring pixels $\left\{g_{p}|p=0,\ldots ,P-1\right\}$. In Equation (3), LBP computes a pixel value from a 8−bit string from the 3×3 neighborhood by computing the Heaviside function t(·) of the difference between neighboring pixels and the central pixel, $\left(g_{i}-g_{c}\right)$ (Figure 1).

LBP-like texture descriptors have evolved into almost all fields of computer vision, because of their robustness to monotonic gray-scale changes, illumination invariance, and computational simplicity. Invariance w.r.t. any monotonic transformation of the gray scale is achieved by considering in (Equation (3)) the signs of the differences $t(g_{i}-g_{c}),i=0,\cdots ,P-1$. The local texture can be represented as a joint distribution of the values of the differences at the center pixel $g_{c}$. Assuming the *independence* of $g_{c}$ with respect to the differences $(g_{i}-g_{c}),i=0,\cdots ,P-1$. However, under certain circumstances such as very low or high values of $g_{c}$, the range of possible differences and so, LBP can miss the local structure as it does not consider the central pixel. To reduce the noise sensitivity, mostly in uniform regions, a three-level operator has been proposed by Tan and Trigg [23], which describes a pixel relationship with its neighbors by a ternary encoding, i.e., −1,0,1 rather than a binary code, i.e., 0,1. The size of this code is reduced by splitting it into two LBP (Positive and Negative) codes, which results into two 8-bit strings thus needing a 16 bit space for representation.

In the next section, an algorithm is proposed for generating Z images, which could be utilized in contour detection or image segmentation.

## 3. Z Representation

### 3.1. Zeckendorf Additive Partition

In this section, an algorithm is proposed for so called Z pooling. In [24] the Belgian mathematician Édouard Zeckendorf states that any integer *N* may be *uniquely* represented as the sum of distinct Fibonacci numbers so that the sum does not include any *two non-consecutive* Fibonacci numbers. The Fibonacci series 1,1,2,3,5,8,⋯ is a sequence of numbers f(n) such that f(n) is the sum of the 2 previous values with initial conditions f(0)=f(1)=1:

(4) f(n)=f(n−1)+f(n−2)forn≥0.

Here we have a second-order linear constant coefficient difference equation that we want to solve. Specifically, consider the following by rewriting it in a slightly different form:

(5) f(n)−f(n−1)−f(n−2)=δ(n−1)

The solution to the Equation (4) may be found using z-transforms as follows: $F\left(z\right)-z^{-1}F\left(z\right)-z^{-2}F\left(z\right)=z^{-1}$. Solving for F(z) we have $F\left(z\right)=\frac{z^{-1}}{1-z^{-1}-z^{-2}}$.

**Theorem** **1** (Zeckendorf’s additive theory)**.** *Any positive integer N can be expressed as a sum of distinct Fibonacci numbers (f(1),f(2),f(3),⋯,f(m) with appropriate coefficients $\sigma_{i}\in \left\{0,1\right\}$ such as*

(6) $$N=\sum_{i=0}^{m}\sigma_{i}f\left(i\right).$$

*such that $\sigma_{i}\sigma_{i+1}=0,\;i=1,2,\cdots$.*

**Proof.**  For any positive integer *N*, there is always a positive integer *m* such that f(m)≤n≤f(m+1). If n≠f(m),

(7) 0<N−f(m)<f(m+1)−f(m)=f(m−1).

Since N−f(m) is positive, there exists a positive integer *p* such that

(8) f(p)≤N−f(m)<f(p+1).

Now f(p)≤N−f(m)<f(m−1) implies p≤m−2, i.e., f(p) and f(m) are not consecutive Fibonacci numbers. If N−f(m)≠f(p), there exists a positive integer q≤p−2 such that

(9) f(q)≤N−f(m)−f(p)<f(q+1)

and the process continues. Ultimately, we must reach the point where the partial sum equals a Fibonacci number—say f(t)—and thereby obtain the desired representation

(10) N=f(m)+f(p)+f(q)+⋯+f(t).

 □

Zeckendorf partition is *complete* and *canonical*, i.e., every positive integer is the sum of distinct elements of Fibonacci series and, in the binary base, the sequence $\sigma_{k},\sigma_{k-1},\cdots \sigma_{3},\sigma_{2}$ with $\sigma_{i}\in \left\{0,1\right\}$ in Equation (6) contains the smallest number of 1. The number of Fibonacci sequences of length k−1 is exactly f(k+1).

An 8-bit gray scale image has the intensity values in the range [0,255]. The first Fibonacci numbers below 255 is the discrete set F={1,2,3,5,8,13,21,34,55,89,144,233} of cardinality |F|=12. So the Fibonacci sequence can be used for 12-bit image coding and each pixel intensity of an image can be encoded as a sum of distinct consecutive or non-consecutive Fibonacci numbers. For instance the pixel value 255 can be represented by the sequences ${\left(1,21,233\right)}_{\mathrm{fi}}$ ${\left(1,8,13,233\right)}_{\mathrm{fi}}$ ${\left(1,21,89,144\right)}_{\mathrm{fi}}$ ${\left(1,3,5,13,233\right)}_{\mathrm{fi}}$ ${\left(1,8,13,89,144\right)}_{\mathrm{fi}}$ ${\left(1,21,34,55,144\right)}_{\mathrm{fi}}$ ${\left(1,3,5,13,89,144\right)}_{\mathrm{fi}}$ ${\left(1,8,13,34,55,144\right)}_{\mathrm{fi}}$ ${\left(1,3,5,13,34,55,144\right)}_{\mathrm{fi}}$ but Zeckendorf decomposition ${\left(233,21,1\right)}_{\mathrm{Zck}}$ is unique. bit patterns.

From this additive property of integers, a new image encoding is proposed (see Algorithm 1), which encodes the local dependencies of pixels by combining a pooling operation and an integration operation, both chosen from supremum (max), infimum (min), summation, intersection (∩) or set difference (∖) [25]. A *texel* is a texture element or texture pixel.

The way these operators are combined results in images that could be directly used in the computer vision pipeline for object segmentation or contour extraction. The result of applying various arithmetic operations after the intersection leads to different types of image variations.

**Figure 2:**
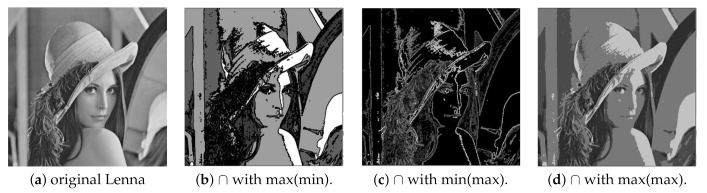
Z-images resulting from the application of Algorithm 1 on Lenna picture by combining ∩ with four arithmetic operations from top left clockwise.

Four of these variations on Lenna’s image are shown on Figure 2. Each produces a characteristic inference line, which we explore below.

The first Figure 2a is produced by applying the supremum operation followed by another supremum. The edges are quite smooth and many edges are missed due to the maximum operation. This operation leaves smaller values in the intersection, resulting in fewer or no edges. Figure 2b is constructed by applying the supremum operation followed by an infimum. As expected, the max operator at the initial stage will produce the set of relatively larger values leaving small Fibonacci numbers. A minimum operator at the end slightly overcomes the maximum effect by selecting the minima for the central pixel. Figure 2c could be considered the complete opposite of the second. All the minimum values are first extracted using the infimum operator, then the supremum of the set is taken. It is totally intuitive to think of it as a double of the second image. Figure 2d is produced by applying a summation operator, which is then followed by the minimum operation. The difference between the fourth and the second images is that the values are out of range for some pixels due to the intensity ranges saturating the summation operator.
**Algorithm 1:** Image Z coding.
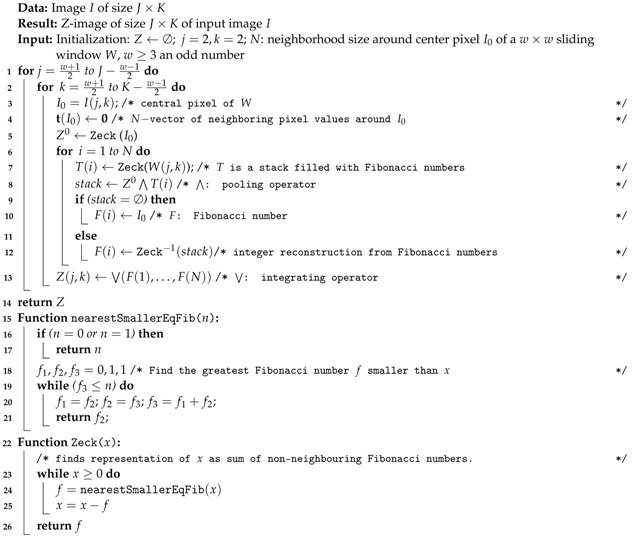


In Algorithm 1, e.g., for w=3, the list of neighbor pixels surrounding $I_{0}$ is W(j,k)=[I(j−1,k−1)I(j−1,k)I(j−1,k+1)I(j,k+1)I(j+1,k+1)I(j+1,k)I(j+1,k−1)I(j,k−1)].

**Example** **1** (Z coding)**.** *Consider a pixel $I_{0}$ of intensity 183, surrounded by the eight neighbor pixels of values $t={\left(210,106,231,233,79,142,209,188\right)}^{T}$.*

*The Zeckendorf decomposition T of the neighbor pixels are respectively ${\left(144,55,8,3\right)}_{\mathrm{Zck}}$${\left(89,13,3,1\right)}_{\mathrm{Zck}}$${\left(144,55,21,8,3\right)}_{\mathrm{Zck}}$${\left(233\right)}_{\mathrm{Zck}}$${\left(55,21,3\right)}_{\mathrm{Zck}}$${\left(89,34,13,5,1\right)}_{\mathrm{Zck}}$${\left(144,55,8,2\right)}_{\mathrm{Zck}}$${\left(144,34,8,2\right)}_{\mathrm{Zck}}$ and for the central pixel $Z^{0}={\left(144,34,5\right)}_{\mathrm{Zck}}$. Then $T\left(1\right)={\left(144,55,8,3\right)}_{\mathrm{Zck}}$, $T\left(2\right)={\left(89,13,3,1\right)}_{\mathrm{Zck}}$, etc.*

*Following Algorithm 1, consider first the pooling operation ∩ (line 10) applied to the center pixel and the first neighbor pixel. Then $Z^{0}\cap T\left(1\right)={\left(144,34,5\right)}_{\mathrm{Zck}}\cap {\left(144,55,8,3\right)}_{\mathrm{Zck}}={\left(144\right)}_{\mathrm{Zck}}$. Therefore, F(1)=144.*

*Similarly for the second pixel $Z^{0}\cap T\left(1\right)={\left(144,34,5\right)}_{\mathrm{Zck}}\cap {\left(89,13,3,1\right)}_{\mathrm{Zck}}=\emptyset$. Therefore, $F\left(2\right)=I_{0}=183$.*

*Finally, for the last pixel ${\left(144,34,8,2\right)}_{\mathrm{Zck}}$, $Z^{0}\cap T\left(8\right)={\left(144,34,5\right)}_{\mathrm{Zck}}\cap {\left(144,34,8,2\right)}_{\mathrm{Zck}}={\left(144,34\right)}_{\mathrm{Zck}}$. If one choose suppremum operator, then F(8)=max(144,44)=144.*

*After the stack F is populated with the Fibonacci values, $F={\left(144,183,144,183,183,34,144,144\right)}^{T}$. the supremum of this set is calculated (line 15) and treated as the Z-code, which replaces the central pixel value 183 in this example.*

**Figure 3:**
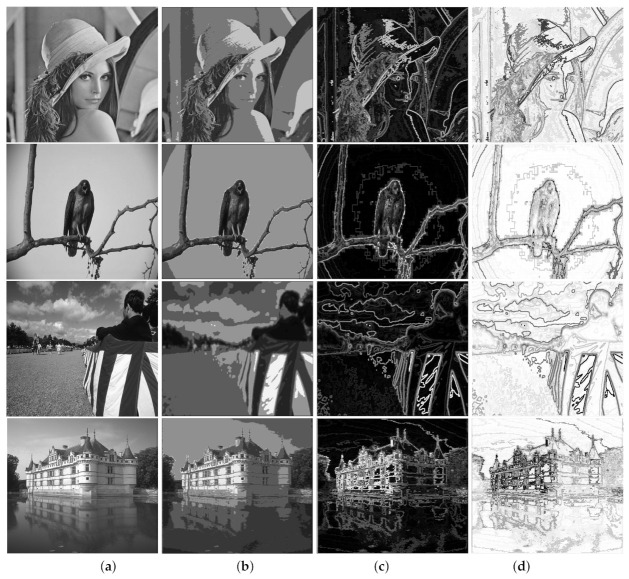
Z-coded images. (**a**) original images. Segmented images obtained by combining intersection with (**b**) max(max) (**c**) min(max) (**d**) max(min).

Figure 3 shows image variations on other types of pictures.

### 3.2. Evaluation and Result for Segmentation

Local image descriptors perform well on various computer vision tasks such as image retrieval [26], action recognition [27,28], object detection and recognition [29] etc. We discuss the Zeckendorf representation as a local image descriptor for two of these tasks.

Algorithm 1 results in ultrametric contours or segmented images based on the association of the aforementioned operations.

The union operator was not included in this work because computer vision generally derives directly from the ability of image descriptors to be discriminating, and this is achieved by intersection or set difference operators.

Table 1 reports the performances of the top 10 algorithms and the Zeckendorf segmentation on 500 test images of BSD500 [30], combining set difference and max(max) operators. Segmented images obtained after region merging were also compared with the human annotated images using the benchmark code available at Berkeley’s website in Table 2 [30]. We evaluated the quality of the extracted boundaries using Precision and Recall measures. Here, the Precision *P* is the probability that the extracted borderline pixel is a true borderline pixel and Recall (sensitivity) *R* is the probability that the real borderline pixels are correctly extracted:

(11) $$P=\frac{TP}{TP+FP}\;R=\frac{TP}{TP+FN},$$

where TP,FP and FN are resp. the true positives, the false positives, and the false negatives. Precision is how sure one is of true positives whilst Recall is how sure one is about not missing any positives. Due to the trade-off between the two mentioned measures, we calculated an *F*-score from Equation (11) to compare the results obtain after regions are merged:

(12) $$F=\frac{PR}{\alpha P+(1-\alpha )R}$$

with α an adjustable perimeter, selected here as 0.5 to compare our results with results available from other algorithms. The *F*-measure of Z coding is 0.6652 with an average Recall of 0.833 (the highest), indicating that the edge pixels are rarely misclassified. This *F*-score could be further improved by refining certain factors such as postsegmentation region-fusion procedures.

**Figure 4:**
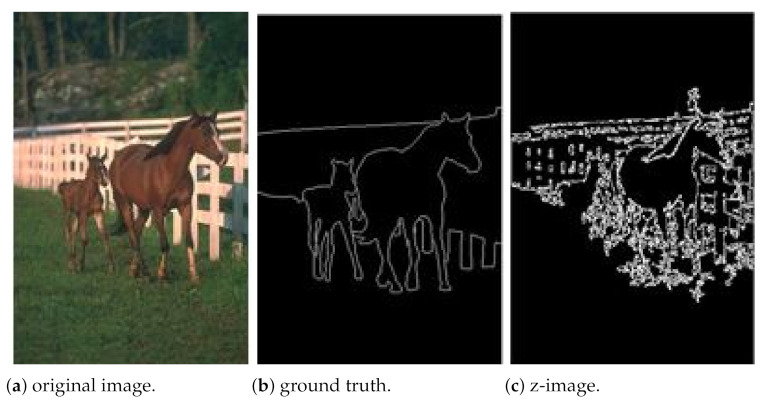
Z-coded color images using Zeckendorf representation. (**a**) original images (**b**) ground truth image (**c**) binary segmented images obtained with set difference and min(max) operators. Precision: 0.5833. Recall: 0.7871. *F*-Score: 0.6700.

Figure 4 illustrates the calculus of the performance metric of the segmentation process on the “horse” image from BSD500.

## 4. Z Pooling

Let *h* be the input volume (or image) with axis sizes $n_{k}$ and *g* the convolution kernel with axis sizes $m_{k}$. In CNN the channel or feature axis has a special role. By convention *g* is the identity along the feature axis and the output of the convolution can have multiple features. The number of input features is $n_{N}$, given by *h*. The number of output features is $n_{e}tal.pha$ and a parameter of the convolution operation. This is achieved by packing multiple convolutions into one operation, one for each output feature. The set of valid indices for the input volume *h* is defined as $A=\{\left(i_{1},i_{2},\cdots ,i_{N}\right)|i_{k}\in \left[1,\cdots ,n_{k}\right],k\in \left[1,\cdots ,N\right]\}$. As there are multiple output features the kernel *g* gets an additional axis and thus the indices of *g* are in the set $B=\{\left(j_{1},j_{2},\cdots ,j_{N+1}\right)|j_{b}\in \left[1\cdots m_{k}\right],b\in \left[1,\cdots ,N+1\right],m_{N}=n_{N},m_{N+1}=n_{e}tal.pha\}$. The resulting volume of the convolution operation has the same axis sizes as *h* except for the feature axis. The convolution with zero padding written in the terms of volumes becomes:

(13) $$\begin{array}{cc} a_{k} & =i_{k}-j_{k}+\left\lceil m_{k}/2\right\rceil \;k\in \left[1\cdots N-1\right] \end{array}$$

(14) $$\begin{array}{cc} a_{N} & =j_{N} \end{array}$$

(15) $$\begin{array}{cc} h_{a_{1}a_{2}\cdots a_{N}} & =0\;\forall (a_{1}a_{2}\cdots a_{N})\notin A \end{array}$$

(16) $$\begin{array}{cc} {\left(h*g\right)}_{i_{1}i_{2}\cdots i_{N}} & =\sum_{j_{1}j_{2}\cdots j_{N}}h_{a_{1}a_{2}\cdots a_{N}}g_{j_{1}j_{2}\cdots j_{N}} \end{array}$$

with * is the convolution operation and ⌈·⌉ the ceiling function. In many publications the kernel size is split into a image and feature part, i.e., the convolution operation defined by Equation (13) would be described as a convolution with a $m_{1}\times m_{2}\times \cdots m_{N-1}$ kernel and $m_{N}$ input features/channels and $m_{N+1}$ output features/channels. The number of input features is determined by the (known) size of the input image *h* and thus almost always omitted.

Maximum pooling is an important operation for contemporary neural networks defined for a input volume (or image) $h:A\to R,\;A=\{\left(i_{1},i_{2},\cdots ,i_{N}\right)|i_{k}\in \left[1,\cdots ,n_{k}\right],k\in \left[1,\cdots ,N\right]\}$ and a set $B=\{\left(i_{1},i_{2},\cdots ,i_{N-1},0\right)|i_{b}\in \left[-K_{b},\cdots ,K^{b}\right],b\in \left[1,\cdots ,N-1\right]\}$ called window where either $K_{b}=K^{b}$ or $K_{b}=K^{b}-1$ with $K^{b}\in N$:

(17) $$\max\mathrm{pool}\left(h,B\right)\left(x\right)=\max_{y\in B}h\left(x-y\right).$$

The *x* and *y* are indices for the volume *h* and *B* can be seen simply as a selection mask. Note that this operation looks for the maximum in a neighborhood defined by *B* along the image axis. Unlike the convolution the channels are not mixed in this operation. Often the maximum pooling operation is used for downsampling the volume by restricting *x*. This restriction is called *striding* with stride s∈N and *A* is restricted to $A^{\prime }=\left\{\left(i_{1},i_{2},\cdots ,i_{N}\right)|i_{k}\in \left[1,1+s,1+2s,\cdots ,1+n_{s}s\right],n_{s}=\left\lceil n_{k}/s\right\rceil -1,k\in \left[1\cdots N\right]\right\}$. The strided max pooling operation is then:

(18) $$x^{\prime }=1+sx,\;\max\mathrm{pool}\left(h,B,s\right)\left(x\right)=\max_{y\in B}h\left(x^{\prime }-y\right).$$

The strided max pooling reduces the size of the input image by only considering every *s*-th entry along all image axes and discarding all others. The concept of strides can be used for convolution operations as well and where fractional strides can even be used for upsampling [32].

Z pooling can easily replace maximum pooling in a CNN in Equation (17) when writing

(19) $$Z\;\mathrm{pooling}\left(h,B\right)\left(x\right)=\max_{y\in B}f\left(x⋀Zck\left(y\right)\right)),$$

where ⋀ is the intersection or set difference operation. *B* is the mask (neighborhood) in which *x* is selected. Note that Z pooling is an operator without parameters as well as max pooling.

With respect to fully connected neural networks, CNN are translation invariant. The translation invariance comes from the fact that the convolution kernel *W* is the same for every possible position of the input. So once the network learns to recognize an object in one position on the image it automatically will recognize it at any position. However, the use of convolutions comes with a cost: the number of parameters grows with the input and output size. Different pooling operations were carried out in a categorization context to compare the behavior of the different pooling operations.

The most relevant question at this stage is: are pooling layers more efficient when they pool texels or when they pool pixels? Experiments proposed in the following section give some answers.

## 5. Numerical Evaluation with CNN

### 5.1. Implementation

The experiments using the aforementioned algorithms were implemented in Python© 3.7 using Tensorflow and Keras frameworks except for the cascaded network for which the authors provide an implementation based on Niftynet [33]. Computation were completed on a Tesla VT100 CPU @ 3.60 Ghz with 64 GB of RAM. This study focuses on the use of a magnetic resonance imaging (MRI) dataset of acquired brain tumors from the challenge of multimodal brain tumor segmentation Brain Tumor Segmentation (BraTS) challenge [34].

The BraTS publicly available dataset contains preoperative MRI scans for 285 patients. The database is divided into two categories: High-Grade Gliomas (HGG) (210 patients) and Low-Grade Gliomas (LGG) (75 patients). Four MRI modalities of each scan are presented: native (T1), postcontrast T1-weighted (T1Gd), T2-weighted (T2) and T2 Fluid Attenuated Inversion Recovery (FLAIR). The ground truth segmentation is provided (manual segmentation validated by one-to-four experienced neuroradiologists).

### 5.2. Miccai BraTS Dataset

Segmentation of brain tumors from multiple modalities can produce a prediction that facilitates surgical planning, postoperative analysis and radiotherapy [35].

Brain tumors require early detection and sometimes prolonged treatment. They can be benign or malignant when they have a faster growth rate, although benign tumors are slower in growth and include low-grade variants (1–4). Lower grade glioma (LGG) have a higher life expectancy and do not require immediate treatment. Both cases still require neuroimaging prior, during and after treatment. Medical imaging helps to assess tumor progression, surgical planning, and overall treatment [34]. Glioblastoma (GBM) is a very aggressive grade-4 brain tumor, the deadliest among cancers with a five-year survival rates of only 7%.

**Figure 5:**
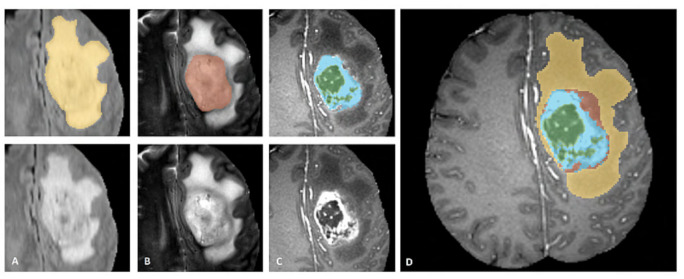
BraTS task description [36]. The whole tumor is visible in FLAIR (**A**), the tumor core in T2 (**B**), the enhancing tumor structures in T1c (blue), surrounding the cystic/necrotic components of the core (green) (**C**). Combined segmentation give the final labeled image (**D**): edema (yellow), nonenhancing solid core (red), necrotic/cystic core (green), enhancing core (blue).

BraTS challenge requires not only the segmentation of the whole tumor but also subsequently the tumor core and enhancing tumor (Figure 5). The Dice coefficient is used to measure the quality of the segmentation.

### 5.3. Experiment Details

In the first experiment we consider 2D U-Net, 3D U-Net, and Cascaded Network for which the training details are presented in Table 3.

The second experiment combines the best method in terms of the highest Dice (i.e., 2D U-Net) with the proposed enhancement methods. Hence, the results of the retrained model are presented following a curricular learning (CL) and data augmentation (DA). The third experiment considers the equally weighted majority voting performed using the 3D U-Net, Cascaded Network and the best performing model (i.e., 2D U-Net + CL) from the second experiment. When used, all the DA and CL transformation are applied on 25% of the initial training dataset.

Curricular learning was first proposed by Bengio [37] to deal with nonconvex optimization to avoid the local optimum issue. The intuition behind Curricular Learning is to mimic the learning of human with a gradual training process with examples sorted in an increasing level of difficulty. Following this idea, we propose to pretrain the considered models from artificially downsampled MRIs by a progressive increasing level of resolutions. This enhancement was carried out by downsampling then upsampling by successive factors equal to eight, four and two. Hence, the first model is trained with the data that is downsampled/upsampled by a factor eight. Once saved, it is retrained with the data that is downsampled/upsampled by a factor four. This process is then repeated with the data downsampled/upsampled by a factor two. Finally, the resulting model is trained with the data in its original resolution.

Data augmentation is used to improve the robustness of the model by artificially increasing the size of the training dataset. In this study, the following geometrical transformations are used with randomly chosen settings: (a) 90 degrees rotation, (b) Horizontal/vertical flip, (c) Cropping, (d) Gaussian white noise.

In order to simultaneously take benefit of all the investigated methods, this proposal consists in developing an original method which combines the predictions provided by each technique (i.e., 2D U-Net, 3D U-Net, Cascaded network). An equally-weighted majority voting is then applied to each pixel of the input MRI. For the prediction, all the methods have the same relevance (weight) to assign a score to each prediction. The final decision is set to use the prediction, which obtains the highest voting score. If several different predictions obtain an identical score, the final prediction is randomly chosen among the best proposed choices.

#### 5.3.1. Data

BraTS dataset was split using 125 patients: 25 patients are used for test, 75% for training, 25% for validation. To improve the computation efficiency of our evaluation, each mri of the dataset was cropped from 240 × 240 × 155 to 144 × 160 × 60, removing background region pixels.

#### 5.3.2. Training Protocol

All the three supervised methods were trained using the Dice Loss Function (DLF) equal to one minus the Sørensen–Dice index:

(20) $$\mathrm{DLF}\left(P,T\right)=1-\frac{2\sum_{i}P_{i}\times T_{i}}{\sum_{i}P_{i}+\sum_{i}T_{i}+\epsilon }$$

where *P* denotes the set of the predicted pixels ($P_{i}$ being the *i*-th element) and *T* the set of the corresponding ground truth. We arbitrary defined in Equation (20) ϵ=1 to deal with the particular case when *P* and *T* only contain background values equal to zero. The 2D and 3D U-Net were trained with 300 epochs while the Cascaded Network was only trained for 30 epochs due to time constraints. The network requires separate training for each region and each of the three views, which increases training time.

**Figure 6:**
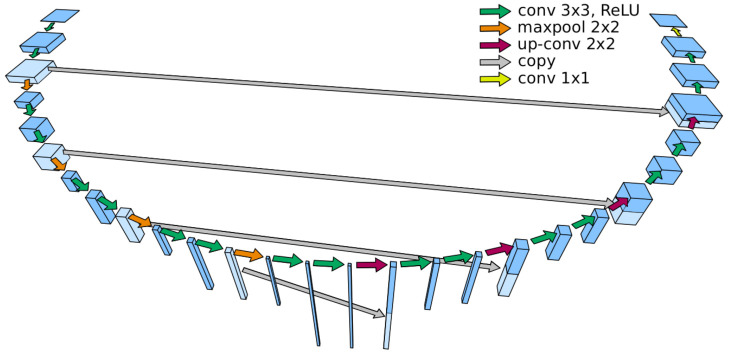
U-Net architecture with its encoder and decoder structure. The arrows represent the operations and the volumes are the characteristics: the height of the volume corresponds to the number of feature maps and the width and depth of the volume for the size of the feature maps. This U-Net uses 5 different resolutions.

2D U-Net was first proposed for biomedical image segmentation by Ronneberger et al. [38] (Figure 6). This architecture contains two paths respectively called encoder and decoder, which contain several convolutional and maximum pooling layers at the encoder level and transposed convolution (up-conv) layers at the decoder. The autoencoder is designed to find a latent representation of a dimension smaller than the input that is used for the segmentation task. Unlike the U-Net originally proposed, zero-padding is used as well rather than maximum pooling to preserve the dimension of the output at each layer, allowing more flexibility for the dimension of the input. The U-Net used in this article follows the U-Net architecture proposed by Dong et al. [39] depicted in Figure 6.

3D U-Net extends the U-Net network for volumetric segmentation [40]. The input is taken as the voxels of the volumetric images and the resulting output is a 3D segmentation mask. All the operations are in 3D and a batch normalization of 10 has shown to improve the training convergence. Another difference is the reduction in the number of blocks in each path from five to four. The Dice loss function Equation (20) was also used for the training of this network. The encoder path contains two 3D convolutions followed by a Rectified Linear Unit (ReLU), and a 2 × 2 × 2 maximum pooling with strides of two. The decoder path blocks include 2 × 2 × 2 transposed convolution (up-conv) by strides of two in each dimension and two 3D convolutions followed by ReLU. The entire image is analyzed in the contracting path and subsequent extensions produce the final segmentation.

**Figure 7:**
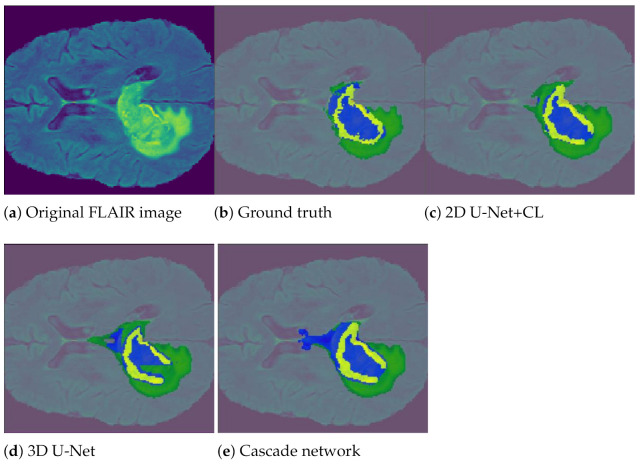
Brain tumor segmentation of a MRI slice using three different methods (**c**–**e**). Given the original FLAIR image in (**a**), the different sub-regions correspond to predicted whole tumor region (green+yellow+blue), tumor core region (yellow+blue), enhancing core (blue). Ground truth image (**b**) is obtained with manual segmentation.

Cascade network proposed by Wang et al. [33] includes a combination of three CNNs that segment each of three subregions sequentially: whole tumor, tumor core and enhancing tumor. Hence, anisotropic convolution (i.e., dependent on the direction) are used to deal with 3D MRI but it results in a higher model complexity and memory consumption. Lastly the fusion of the CNN outputs in three orthogonal views: axial, sagittal, and coronal is used to enhance the segmentation of the brain tumor. The three CNNs follow the hierarchical structure of the tumor subregions as depicted in Figure 7.

After the convolutional layer with zero padding, we get feature maps of the same size as the input. Then each feature map is passed through Z pooling with stride one and *k* different windows of sizes $d_{1}\times d_{1},d_{2}\times d_{2},\cdots ,d_{k}\times d_{k}$ are used. The second layer is responsible for the increase of the receptive field, which is determined by the larger window size $d_{k}$. For an input of size s×s we suggest $d_{k}=2s$ to ensure that the receptive field is as large as the input image. In these experiments, the multiplicity is chosen at m=10 and the window size $d_{i}=2^{i-1}+1$ i.e., $d_{i}\in \left\{1,3,5,9\right\}$. This is a good compromise between the size of the network and the expected performance. Hence $k=\lfloor \log_{2}\left(s\right)+2\rfloor$. The other window sizes determine the scales for which the information is collected. The initial convolution ensures that the features are relevant for each scale. The multiplicity *m* makes it possible to collect multiple features by scale. The convolution layers are followed by ELU [41] as an activation function.

#### 5.3.3. Results and Discussion

Results presented in Table 4 (and illustrated in Figure 7) show the effectiveness of each method measured in terms of Sørensen–Dice index, Recall and Precision only on the tumor core region, the most difficult to segment. The pooling layers with configuration-2 favor segmentation unlike configuration-3, which favor ultrametric contours.

According to Table 4, the 2D U-Net obtains the highest Dice scores for the three subregions during the first experiment: tumor core = 0.65 (and for the record whole tumor = 0.65 and increased tumor = 0.46). The scores for the Cascade and 3D U-Net are not drastic. Given this result, the 2D U-Net was chosen for the improvement experiences: CL and DA. In regard to the equally weighted majority vote, the prediction of the first three methods was used to obtain the final prediction. The improvement in 2D U-Net results shows that the three improvement methods proposed improve Dice score and Precision, but the 2D U-Net formed with CL outperforms the others. Z pooling works comparatively better than maximum pooling in terms of accuracy, Recall, and Dice score. The Dice score indicates that Z pooling with max(min) misses fewer tumor cores on average than the max(max) combination. The best combination is obtained with a 2D U-Net with Curricular Learning, Zeck min(max) (Dice = 0.77, Recall = 0.8, Precision = 0.87) while comparatively 2D U-Net alone gives lower scores (Dice = 0.72, Recall = 0.79, Precision = 0.77). Note that the 2D U-Net association with DA and CL provides disappointing performances.

The intuitive explanation is that Z-pooling prepares CNN better than maximum pooling for the segmentation tasks by sharpening the edges. The weights of the CNN shall accentuate the edges during training whenever there is a significant difference between two adjacent pixels. In capturing ultrametric contours Z-pooling can be seen as a kind of pretraining of the network to accelerate the learning and enhance the segmentation result.

## 6. Conclusions

To conclude, the experiments presented along with the results have demonstrated a pipeline of evaluation for the supervised segmentation MRI images with Z pooling. CNNs have once again proven to excel in image processing and more specifically in learning and distinguishing characteristics that then enables segmentation. Simple or complex addition or changes based on Z pooling have proved to improve results, which reinforces the need to further advance research in this area. The goal of this research was met, which was not only to examine the presented methods but also to introduce the enhancements and enable a thorough comparison.

Earlier, we raised two questions: when should we perform pooling? Is texel pooling more efficient than pixel pooling?

It is advisable to pool when we can extract features contained in the binned subregions from the input representation (input image, hidden layer, etc.) As mentioned in the discussion, some of the enhancements in the pooling improved certain results and diminished others. However, in most of our experiments, the texelization of the pooling layer improved the image segmentation capacity of the CNN. It is because Z coding, compared to other local descriptors: (a) can be extended to any neighborhood size or geometry, (b) is invariant in shift (c) is invariant in rotation, (d) is nonlinear, (e) follows a integer generating function, (f) is less sensitive to noise.

The correct scale is therefore part of the definition of texture and plays an important role. In other words, texel pooling is more efficient in general because our world is “textured” but performance decreases directly as signal-to-noise ratio gets worse.

We challenged the concept for feature extraction, which has been uncontested for three decades, the feature extraction pyramid. Our method translates the series of solutions to enhance Z pooling with different window sizes. The effective receptive field of our method can be modified freely through the pooling window sizes without affecting the parameter number, whereas traditional feature extraction pyramids have a high parameter cost associated with an increase of the receptive field.

Further investigations should target all combinations of Z pooling operators and find out a performance criterion to maximize that describes the pixel organization.

## Figures and Tables

**Table 1:** Comparison of Z coding with 10 top-ranked segmentation algorithms for the BSD500 benchmark using set difference and max(max) operators. See [31] for a review.

Rank	Algorithm	Average Recall	Average Precision	Average *F*-Measure
1	gPb-UCM	0.7397	0.7241	0.7226
2	Global Probability of Boundary (GPB)	0.7261	0.6902	0.7031
3	Ren	0.7198	0.6959	0.7019
4	**Z-coding**	**0.833**	**0.5875**	**0.6652**
5	Brightness/Texture Gradients (BTG)	0.6999	0.637	0.6592
6	Boosted Edge Learning (BEL)	0.699	0.6254	0.6557
7	Brightness Gradient (BG)	0.6946	0.6011	0.6348
8	Multiscale Gradient Magnitude (MGM)	0.6562	0.5939	0.6133
9	Gradient Magnitude (GM)	0.6961	0.5677	0.6119
10	Texture Gradient (TG)	0.6231	0.6053	0.6076
11	Second order Moment Matrix (SMM)	0.6501	0.5891	0.6042

**Table 2:** Performance metrics when using Z coding versus human segmented images on BSD500.

Indicators	Average Recall	Average Precision	Average *F*-Measure
Proposed vs. manual benchmark	0.8326	0.2495	0.3675

**Table 3:** Experiment Details.

Method	Loss Function	Training Set Size	# of Trainable Parameters	Test Set Size	Epochs	Training Duration (Hours)
Cascaded Network	Dice Loss	100	n/a	25	30	9
2D U-Net	Dice Loss	100	31,032,451	25	300	13
3D U-Net	Dice Loss	100	14,491,619	25	300	24

**Table 4:** Comparative results for segmentation of tumor core. Pooling layer choice: ① = (regular) maximum pooling ② = Zeck ∩ followed max(max) ③ = Zeck ∩ followed min(max).

DL Network	Dice Score			Recall			Precision		
Z Pooling Config	①	②	③	①	②	③	①	②	③
Cascaded Network	0.58	0.71	0.52	0.73	0.71	0.76	0.80	0.88	0.88
2D U-Net	0.65	0.62	0.72	0.77	0.77	0.79	0.81	0.86	0.77
3D U-Net	0.46	0.55	0.46	0.72	0.73	0.75	0.86	0.83	0.84
2D U-Net + CL	0.68	0.73	0.77	0.77	0.76	0.80	0.80	0.81	0.87
2D U-net+DA	0.67	0.75	0.62	0.77	0.76	0.82	0.81	0.83	0.78
2D U-Net + DA + CL	0.65	0.68	0.53	0.78	0.77	0.80	0.82	0.90	0.88
Majority Voting	0.61	0.67	0.60	0.76	0.75	0.79	0.82	0.85	0.84

CL = Curricular Learning, DA = Data Augmentation

## Data Availability

BraTS dataset can be downloaded at URL https://www.med.upenn.edu/sbia/brats2017/data.html (accessed on 12 December 2020). Python code can be found at https://github.com/ikramabdel/tumorsegmentation (accessed on 12 December 2020).
